# Early Versus Delayed Norepinephrine Initiation in Septic Shock: A Systematic Review and Meta-Analysis of Randomized and Observational Studies

**DOI:** 10.7759/cureus.98694

**Published:** 2025-12-08

**Authors:** Chibuzo C Manafa, Oluwayemisi E Ekor, Akintunde C Akinboboye, Okelue E Okobi, Gift Ojukwu, Osemwegie O Ugbo, Michael U Mochu, Emasenyie Isikwei, Sergio Hernandez Borges, Miguel Diaz-Miret

**Affiliations:** 1 Family Medicine, Queensland Medical Clinic, Calgary, CAN; 2 Medicine, School of Medical Sciences, University of Cape Coast, Cape Coast, GHA; 3 Emergency, University of Medical Sciences Teaching Hospital, Ondo, NGA; 4 Family Medicine, Larkin Community Hospital Palm Springs Campus, Hialeah, USA; 5 Family Medicine, IMG Research Academy and Consulting LLC, Homestead, USA; 6 General Practice, Leeds Teaching Hospital, Leeds, GBR; 7 Family Medicine, University of Nigeria College of Medicine, Enugu, NGA; 8 Internal Medicine/Family Medicine, Larkin Community Hospital Palm Springs Campus, Hialeah, USA

**Keywords:** early norepinephrine, hemodynamic stabilization, meta-analysis, mortality, septic shock, vasopressor timing

## Abstract

Septic shock remains a major cause of illness and death worldwide despite improvements in critical care, and the optimal timing for starting norepinephrine continues to generate debate.

This review assessed whether administering norepinephrine within the first hour of recognizing shock or upon ICU admission provides meaningful advantages compared with delayed initiation.

A broad search of major databases from 2010 to May 2025 identified randomized trials and observational studies examining early versus later administration. Twenty-eight studies met the inclusion criteria, and nine were eligible for meta-analysis.

The pooled results showed that early norepinephrine was associated with a modest but statistically non-significant reduction in mortality (RR 0.90; 95% CI 0.76-1.06; p = 0.18). Observational studies, however, demonstrated a clearer survival benefit, with early initiation linked to a significant decrease in deaths (RR 0.75; 95% CI 0.60-0.94). Moderate heterogeneity (I² = 65.6%) likely reflected variation in study design, patient severity, and differences in defining early treatment.

Overall, the evidence suggests that early norepinephrine may help stabilize hemodynamics more quickly and could improve clinical outcomes, though current randomized data remain limited.

Further high-quality research is needed to better define the magnitude of benefit and guide consistent practice.

## Introduction and background

Septic shock has remained a leading cause of mortality and morbidity, despite the advances in critical care management in the world and the integration of technology in healthcare [1]. Septic shock is formally defined as sepsis with persistent hypotension requiring vasopressors and elevated lactate levels despite adequate fluid resuscitation. It is the most extreme form of sepsis, which is a condition of life-threatening circulatory and cellular abnormalities leading to severe hypotension and hypoperfusion of the tissues [2-3]. The prevalence of sepsis is said to claim close to 11 million lives each year across the world, most of whom succumb to septic shock [4]. Early identification and immediate hemodynamic resuscitation are key survival factors because delayed resuscitation is closely linked with the development of dysfunction in all organs, extended intensive care unit (ICU) hospitalization, and death [5].

Timely fluid resuscitation and vasopressor administration to maintain sufficient mean arterial pressure (MAP) and organ vitality perfusion are the main pillars of the management of septic shock [6-7]. Norepinephrine is the most preferred agent among the vasopressors according to the Surviving Sepsis Campaign Guidelines because it has strong α-adrenergic vasoconstrictive properties and a good safety profile [8]. Nevertheless, the most appropriate time of norepinephrine use is a controversial issue [9]. Conventional resuscitation protocols promote the use of vasopressors after proper fluid loading, whereas new data indicates that early norepinephrine can be beneficial in terms of physiological and clinical advantages with a decrease in time to administration - that would be during the first hour following shock identification or ICU admission [10-11]. In this review, early norepinephrine refers to initiation within the first hour of shock recognition or ICU admission.

A key clinical question is whether clinicians should continue fluid resuscitation before starting vasopressors (‘fluid-first’) or initiate norepinephrine early to prevent prolonged hypotension. The early application of norepinephrine is based on pathophysiological information [12]. Cardiac hypotension can lead to sustained tissue hypoxia, endothelial damage, and multi-organ dysfunction [13]. Norepinephrine can prevent excessive fluid resuscitation, limit the excessive vasodilation, and maintain perfusion pressure when administered early, thus preventing the occurrence of fluid overload and its adverse effects [14-15]. In spite of all these possible benefits, doubts still exist about the practice of using vasopressors early, such as the decreased responsiveness to fluids, ischemic issues, and a lack of knowledge as to how patients should be chosen [16].

Observational studies have generally shown lower mortality with early norepinephrine, while randomized trials have produced mixed or neutral findings, emphasizing ongoing uncertainty. Recent studies, randomized controlled trials (RCTs), and observational cohorts provide good results, where some show better survival and quicker shock response to early norepinephrine, and others do not exhibit superior mortality outcome. These results indicate a possibility of missing the full picture of the existing evidence synthesis to define whether norepinephrine being initiated promptly or delayed has an impact on clinical outcomes in the case of septic shock [17-18].

The objective of this study is to evaluate whether early initiation of norepinephrine (<1 hour from shock recognition or ICU admission) reduces mortality and organ dysfunction compared to delayed initiation. Incorporating the results of various studies, this review aims to offer evidence-based information that would be able to optimize the timing of vasopressor initiation in patients with septic shock, as well as contribute to better patient outcomes.

## Review

Eligibility criteria and search strategies

In this systematic review, the PRISMA 2020 guidelines were adhered to in order to provide methodological transparency and reproducibility [19]. The research was outlined on the PICO framework: Population (adult patients with septic shock), Intervention (early initiation of norepinephrine, less than 1 hour after shock recognition or ICU hospitalization), Comparison (late initiation of norepinephrine), and Outcomes (mortality, organ dysfunction, and shock resolution). Published studies in English during the period 1 January 2010 to May 2025 were included in the peer-reviewed studies. PubMed, Scopus, Web of Science, Embase, and Cochrane Library databases were searched on the combination of MeSH terms and keywords, including: septic shock, norepinephrine, vasopressor timing, early initiation, and mortality, as shown in Table 1. Additional eligible studies were also identified by manually screening the reference lists of the relevant articles and systematic reviews. Only randomized and observational studies that compared early versus delayed initiation of norepinephrine were included.

**Table 1:** Overview of the search strategy

Category	Details
Databases Searched	PubMed, Scopus, Web of Science, Embase, Cochrane Library
Time Frame	January 2010 – May 2025
Language	English only
Search Terms	#1 AND #2 AND #3
#1 (Population)	“septic shock” OR “sepsis” OR “severe sepsis” OR “critical illness”
#2 (Intervention/Exposure)	“early norepinephrine” OR “early vasopressor” OR “early initiation of norepinephrine” OR “vasopressor timing” OR “initial norepinephrine use”
#3 (Outcome)	“mortality” OR “organ dysfunction” OR “shock resolution” OR “treatment outcome” OR “ICU survival”

Inclusion criteria

The studies were eligible provided that they directly compared the early and delayed norepinephrine initiation in adults with septic shock and the outcome was examined in terms of mortality, organ dysfunction, or shock resolution. Eligible studies were randomized controlled trials, prospective or retrospective cohort studies, systematic reviews, or meta-analyses that were published in English between January 2010 and May 2025.

Exclusion criteria

Articles that lacked peer review, abstracts of conferences, editorial articles, case reports, and animal or laboratory research were eliminated. Articles that did not specify the timing of norepinephrine, or those that did not report relevant clinical outcomes such as mortality, hemodynamic response, or adverse events, were also excluded to ensure that only studies providing meaningful data for analysis were included.

Screening process

All of the records were imported to EndNote (Clarivate Plc, London, UK) to remove duplicates. Titles, abstracts, and full texts were separately screened by two reviewers who were eligible to proceed to the next stage based on the check-in of a third reviewer in case of dispute.

Quality assessment

In assessing the methodological quality, suitable instruments were applied based on the study design. Study quality was evaluated using the risk of bias in randomised trials (RoB 2; The Cochrane Collaboration, London, UK) tool for randomized trials [20] and the adapted Newcastle-Ottawa Scale for observational studies [21].

Data extraction

An abstraction form was created to gather important data on each of the studies included, such as author(s), publication year, country, study design, and sample size, among others. Information about the time of norepinephrine initiation (early vs delayed), definitions of an early norepinephrine initiation, primary and secondary outcomes (mortality, organ dysfunction, shock resolution, and the initial ICU length of stay), and key findings were compiled in a systematic manner. Other variables like the dose of vasopressor, the use of fluid resuscitation strategy, and characteristics of the study population were also recorded where possible. Two reviewers independently extracted the data using a predesigned template, and the occurrence of any discrepancies was resolved through discussions or by consulting a third reviewer to provide accuracy and consistency throughout all studies in the review.

Statistical analysis

The pooled effect size was presented as a risk ratio (RR) with 95% confidence intervals (CIs). These were calculated using a random-effects model fitted with the Restricted Maximum Likelihood (REML) estimator to account for between-study variability. Subgroup analyses were performed based on study design (RCTs vs. observational studies), and heterogeneity was assessed using the I-squared statistic (I²) and tau-squared (τ²). All analyses were conducted using R version 4.5.0 (R Foundation for Statistical Computing, Vienna, Austria).

Results

**Figure 1:**
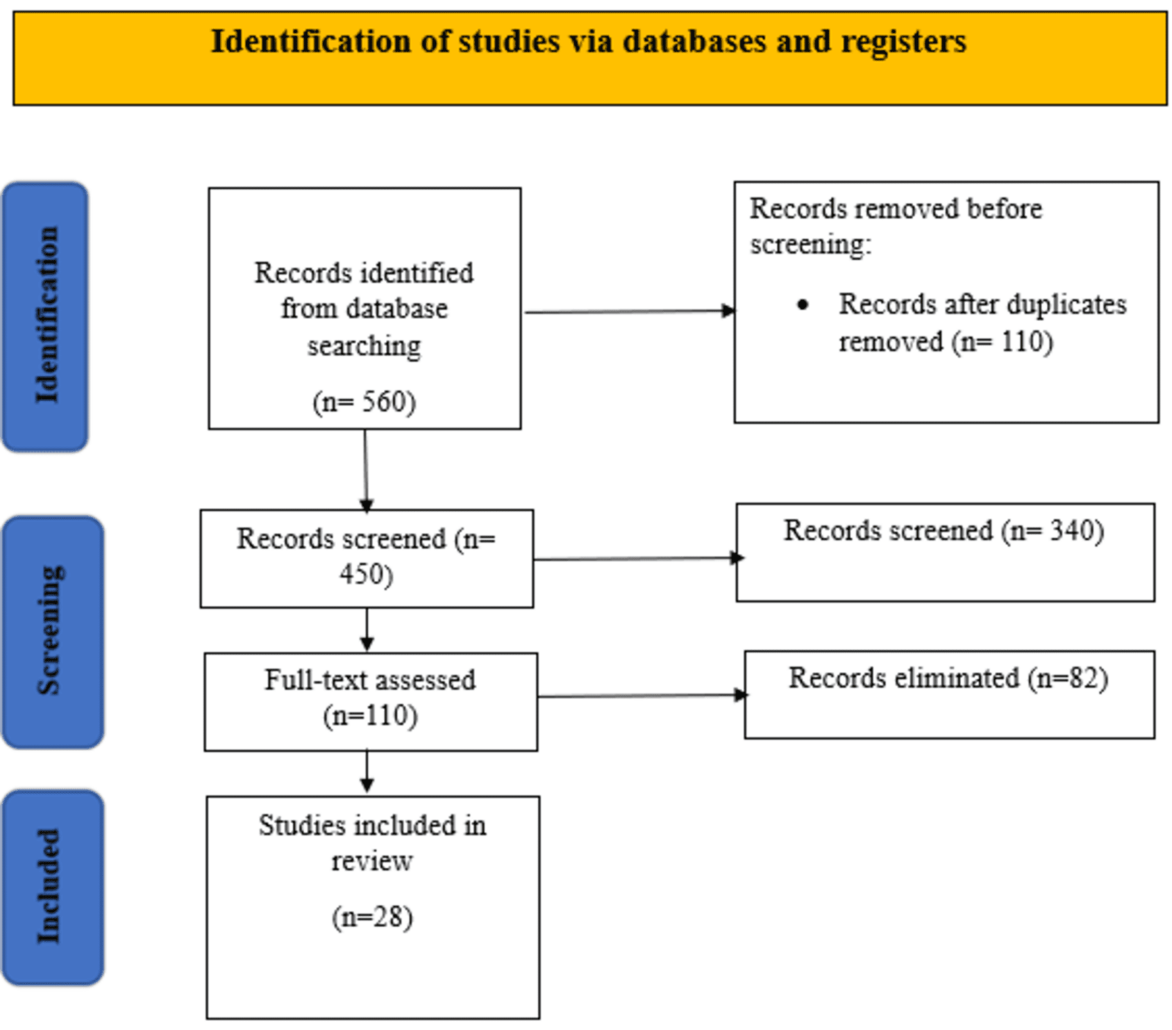
PRISMA flow diagram indicating the study selection and inclusion process This review was conducted in accordance with the PRISMA 2020 guidelines [19]. This work is licensed under the Creative Commons license CC BY 4.0.

A total of 560 articles were obtained after a search of electronic databases. Four hundred and fifty titles and abstracts were filtered after elimination of 110 duplicates. Out of them, 340 studies were filtered out due to their inability to satisfy the inclusion criteria or provide a clear comparison between early and delayed initiation of norepinephrine. The rest 110 full-texts were further evaluated in terms of eligibility, and 82 studies were eliminated due to a lack of related outcomes, lack of definition of the timings, or as non-peer-reviewed materials (e.g., editorials, abstracts of a conference, or a narrative commentary). Finally, 28 studies were included in this meta-analysis and systematic review based on the inclusion criteria as shown in Figure 1. 

In this study, 28 eligible studies were identified for the systematic review. Out of 28 eligible publications, nine studies (six RCTs, one multicenter cohort, and two cohort studies) were extracted for meta-analysis since they reported quantitative data on mortality, as shown in Tables 2, 3. This indicates that, within this study, there are six RCTs and three observational studies based on the design classification. These meta-analysis studies included the total number of patients and mortality events in both the early and delayed norepinephrine groups.

**Table 2:** Summary of the included studies APACHE II: Acute Physiology and Chronic Health Evaluation II; CO: cardiac output; MAP: mean arterial pressure; NE: norepinephrine; OR: odds ratio; SOFA: Sequential Organ Failure Assessment

Study	Country	Design	n (Early)	n (Delayed)	Definition (Early)	Outcome (Mortality)	Main Findings
ProCESS Investigators (2014) [1]	USA	RCT	439	456	Protocol-based early septic shock management	21.0% vs 18.9%	No mortality difference; protocolized care not superior
Vincent JL et al.(2019) [2]	Europe & North America	Systematic review (observational data)	-	-	Meta-analysis of septic shock frequency/mortality	37.3% pooled ICU mortality	High mortality; wide inter-study variability
Hjortrup PB et al.(2016) [3]	Croatia	Prospective observational cohort (single-center, medical ICU)	87	58	Sepsis with persistent hypotension requiring vasopressors to maintain MAP ≥ 65 mm Hg and serum lactate > 2 mmol/L despite adequate fluid resuscitation	ICU mortality ≈ 41% (septic shock ≈ 52% vs sepsis ≈ 23%)	Septic shock was significantly associated with higher mortality; nonsurvivors were older, had higher lactate, SOFA & APACHE II scores
Rudd KE et al. (2020) [4]	Global	Observational (GBD study)	-	-	Global analysis 1990–2017	11 million deaths (2017)	Sepsis remains a leading cause of death globally
Lee SJ et al. (2014) [5]	USA	Cohort (Retrospective)	134	210	<3h fluid resuscitation	Reduced mortality	Early aggressive fluids improved outcomes
Beck V et al. (2014) [6]	Canada	Cohort	285	285	<1h norepinephrine	24.5% vs 35.5%	Earlier vasopressors associated with lower mortality
Hu B et al. (2020) [7]	China	Cohort (Historical)	102	118	Initial high infusion rate	Lower mortality with faster fluids	Faster infusion improved hemodynamics
Ruslan MA et al. (2021) [8]	Malaysia	Observational	-	-	Comparative analysis of norepinephrine	Pooled OR for mortality 0.81	Norepinephrine safe and effective
Bai X et al.(2014) [9]	China	Cohort	81	99	<2h norepinephrine	29.6% vs 43.4%	Improved outcomes with early use
Permpikul C (2019) [10]	Thailand	RCT	155	155	<1h norepinephrine	25.7% vs 34.3%	Lower mortality; faster shock reversal
Ospina-Tascón GA et al.(2020) [11]	Colombia	Cohort (Propensity matched)	186	186	<1h norepinephrine	28-day mortality reduced	Early NE reduced mortality and fluid overload
Adda I et al.(2021) [12]	France	Observational (Physiologic study)	30	30	Early norepinephrine with volume expansion	Improved hemodynamics	Early NE improved preload response
Patel JJ et al.(2017) [13]	USA	Prospective Observational	60	62	<1h after hypotension onset	Lower mortality trend	Prolonged hypotension worsened outcomes
Hamzaoui O et al.(2010) [14]	France	Observational	25	25	<1h norepinephrine	Improved cardiac output	Early NE increased preload and CO
Shi R et al. (2025) [15]	France	Systematic review (Meta-analysis) (Observational)	-	-	Early norepinephrine in septic shock	Neutral pooled mortality	Early NE improved hemodynamics but mortality unchanged
Yeo HJ et al.(2022) [16]	Korea	Registry-based Cohort	320	320	<1h after fluids	Higher mortality (OR 1.3)	Very early NE may increase risk
Ahn C et al.(2024) [17]	Korea	Systematic review (Meta-analysis) (Observational)	-	-	Early vs delayed NE	Mixed mortality effects	Early NE beneficial hemodynamics; unclear mortality benefit
Quenot JP et al. (2013) [18]	France	Cohort (Multicenter)	568	568	Within first hour	High ICU mortality 42%	Early detection improves survival
Hernández G (2018) [22]	Latin America	RCT	212	212	Early goal-directed therapy	43% vs 34.9%	Clinical improvement with early perfusion target strategy
Jeon K et al.(2019) [23]	Korea	Multicenter Cohort	420	460	Sepsis registry early care	30.5% vs 38.1%	Prompt recognition improved survival
Andrews B et al.(2014) [24]	Zambia	RCT	198	202	Modified early goal-directed therapy	64% vs 60%	Feasibility in low-resource setting
Park C et al.(2024) [25]	Korea	Guideline review	-	-	Clinical practice guideline	Not applicable	Summarizes evidence for early NE
Andrews B et al.(2017) [26]	Zambia	RCT	207	206	Early resuscitation protocol	64% vs 67%	No mortality reduction
Jiang Z et al.(2023) [27]	China	RCT	100	100	Restrictive fluid strategy	25% vs 36%	Lower mortality with restrictive fluids
Shi R et al. (2020) [28]	France	Review (Observational)	-	-	Timing and dose of vasopressors	Not applicable	Discusses optimal vasopressor timing
Xu F et al. (2022) [29]	China	Cohort (Propensity score)	187	187	<1h norepinephrine	Lower mortality	Early NE beneficial
Monnet X et al.(2023) [30]	France	Observational	-	-	Personalized early NE	Reduced mortality trend	Supports individualized early NE approach
White KC et al.(2025) [31]	Australia	Target trial emulation	-	-	Adjunct vasopressin <3h	Improved survival	Early vasopressin adjunct reduced mortality

**Table 3:** Risk of bias assessment for randomized controlled trials (RoB 2 tool) Risk of bias for randomized controlled trials was assessed using the RoB 2 tool developed by the Cochrane Collaboration, London, UK [20].

Study (First Author, Year)	Randomization Process	Deviations from Intended Interventions	Missing Outcome Data	Measurement of Outcome	Selection of Reported Results	Overall Risk of Bias
ProCESS Investigators (2014) [1]	Low	Low	Low	Low	Low	Low
Hjortrup PB et al. (2016) [3]	Low	Some concerns	Low	Low	Low	Some concerns
Permpikul C et al. (2019) [10]	Low	Low	Low	Low	Low	Low
Hamzaoui O et al. (2010) [14]	Low	Low	Low	Low	Low	Low
Hernández G et al. (2018) [22]	Low	Low	Low	Low	Low	Low
Andrews B et al. (2014) [24]	Low	Some concerns	Low	Low	Low	Some concerns
Andrews B et al. (2017) [26]	Low	Low	Low	Low	Low	Low
Jiang Z et al. (2023) [27]	Low	Low	Low	Low	Low	Low
White KC et al. (2025) [31]	Low	Low	Low	Low	Low	Low
Park C et al. (2024) [25]	Low	Low	Low	Low	Low	Low

The studies included were randomized controlled trials (RCTs), cohort studies, and retrospective studies, with a combination of clinical settings in North America, Europe, Asia, and Africa. The populations of the studies ranged from single-center ICU cohorts to large multicenter and registry-based studies, providing different ideas on the topic of vasopressor timing and patient outcomes.

Geographically, the immediate use of vasopressor was shown to have similar advantages in the majority of the regions, even though there were some discrepancies in the protocols, availability of resources, and clinical practices. In general, the findings confirm the hypothesis that the early use of norepinephrine in the context of septic shock could render the hemodynamic states more stable, decrease the fluid overload, and lower the mortality rates in the short term, but the results are still incongruent in the context of variability of parameters (study design and the definition of early use).

Figure 1 presents the PRISMA 2020 flow diagram outlining the study selection process from the initial 560 records identified to the 28 studies included in the review. The key study attributes (n=28), including design, sample size, and mortality outcomes, are summarized in Table 2. A summary of the risk-of-bias assessment for all included randomized controlled trials (n=10), conducted using the RoB 2 tool, is provided in Table 3. The quality ratings for all included observational studies (n=18), assessed using the Newcastle-Ottawa Scale (NOS), are presented in Table 4.

**Table 4:** Quality assessment of the observational studies (Newcastle–Ottawa Scale, NOS) Risk of bias for observational studies was evaluated using the Newcastle–Ottawa Scale (NOS) [21].

Study (First Author, Year)	Selection (Max 4)	Comparability (Max 2)	Outcome (Max 3)	Total Score (Max 9)	Quality Rating
Vincent JL et al. (2019) [2]	4	2	3	9	High
Rudd KE et al. (2020) [4]	4	2	3	9	High
Lee SJ et al. (2014) [5]	4	2	3	9	High
Beck V et al. (2014) [6]	4	2	3	9	High
Hu B et al. (2020) [7]	3	2	3	8	High
Ruslan MA et al. (2021) [8]	4	2	3	9	High
Bai X et al. (2014) [9]	3	2	3	8	High
Ospina-Tascón GA et al. (2020) [11]	4	2	3	9	High
Adda I et al. (2021) [12]	3	2	3	8	High
Patel JJ et al. (2017) [13]	3	2	3	8	High
Shi R et al. (2025) [15]	4	2	3	9	High
Yeo HJ et al. (2022) [16]	3	2	2	7	Moderate
Ahn C et al. (2024) [17]	4	2	3	9	High
Quenot JP et al. (2013) [18]	3	2	3	8	High
Jeon K et al. (2019) [23]	4	2	3	9	High
Shi R et al. (2020) [28]	4	2	3	9	High
Xu F et al. (2022) [29]	4	2	3	9	High
Monnet X et al. (2023) [30]	4	2	3	9	High

Meta-analysis findings

Table 5 provides a summary of the nine studies included in the meta-analysis, which includes six RCTs and three observational studies, with the main aim of investigating early versus delayed norepinephrine in septic shock.

**Table 5:** Characteristics of the studies included in the meta-analysis Deaths (Early vs Delayed) represent the absolute number of deaths observed in each treatment group, calculated directly from the reported mortality percentages and total participants per arm.

Study	Design	n (Early)	n (Delayed)	Definition (Early)	Mortality (Early vs Delayed)	Deaths (Early vs Delayed)	Key Findings
ProCESS Investigators (2014) [1]	RCT	439	456	Protocol-based early septic shock management	21.0% vs 18.9%	92 vs 86 deaths	No mortality difference; protocolized care not superior
Beck V et al.(2014) [6]	Cohort	285	285	<1h norepinephrine	24.5% vs 35.5%	70 vs 101 deaths	Earlier vasopressors associated with lower mortality
Bai X et al.(2014) [9]	Cohort	81	99	<2h norepinephrine	29.6% vs 43.4%	24 vs 43 deaths	Improved outcomes with early use
Permpikul C (2019) [10]	RCT	155	155	<1h norepinephrine	25.7% vs 34.3%	40 vs 53 deaths	Lower mortality; faster shock reversal
Hernández G (2018) [22]	RCT	212	212	Early goal-directed therapy	43% vs 34.9%	91 vs 74 deaths	Clinical improvement with early perfusion target strategy
Jeon K et al.(2019) [23]	Multicenter Cohort	420	460	Sepsis registry early care	30.5% vs 38.1%	128 vs 175 deaths	Prompt recognition improved survival
Andrews B et al.(2014) [24]	RCT	198	202	Modified early goal-directed therapy	64% vs 60%	127 vs 121 deaths	Feasibility in low-resource setting
Andrews B et al.(2017) [26]	RCT	207	206	Early resuscitation protocol	64% vs 67%	132 vs 138 deaths	No mortality reduction
Jiang Z et al.(2023) [27]	RCT	100	100	Restrictive fluid strategy	25% vs 36%	25 vs 36 deaths	Lower mortality with restrictive fluids

Table 6 shows the pooled analysis estimates that assessed the association between early versus delayed norepinephrine initiation and mortality among patients with septic shock.

**Table 6:** Summary of the meta-analysis results

Model	Pooled Risk Ratio (RR) [95% CI]	p-value	I² (%)	τ²	95% Prediction Interval
Common-effect model	0.91 [0.84–0.98]	0.016	–	–	–
Random-effects model	0.90 [0.76–1.06]	0.175	65.6	0.030	[0.62–1.29]

The random-effects model revealed a statistically insignificant trend toward lower mortality with early norepinephrine administration (RR = 0.90, 95% CI: 0.76-1.06; p = 0.18). However, the common-effect model reported a modest but statistically significant reduction (RR = 0.91, 95% CI: 0.84-0.98; p = 0.016). This is a clear indication of a potential clinical advantage for early norepinephrine use, though the observed heterogeneity requires cautious interpretation.

**Figure 2:**
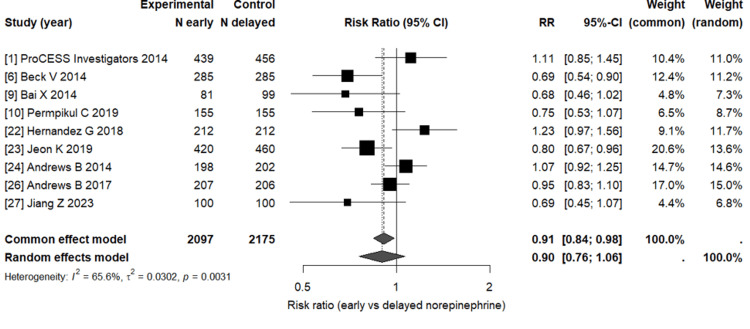
Overall pooled meta-analysis of early versus delayed norepinephrine administration A forest plot showing the individual and pooled risk ratios (RRs) comparing the impact of early versus delayed norepinephrine initiation on mortality. Each square represents a study-specific RR, with the horizontal lines showing 95% confidence intervals. The diamond represents the pooled estimate from the random-effects model.

To visualize the findings presented in Table 6, Figure 2 presents the overall pooled risk ratios across the nine included studies.

As shown in Figure 2, the random-effects model reported a risk ratio that is not statistically significant, indicating a 10% relative reduction in mortality. Further, from the figure, it’s evident there exists a moderate heterogeneity (I² = 65.6%, τ² = 0.03), which is a reflection of variations among studies likely due to design and patient differences. In summary, the findings reveal that early norepinephrine use appears effective in improving the outcomes, but given the inter-study variability, the results should be interpreted with caution.

**Figure 3:**
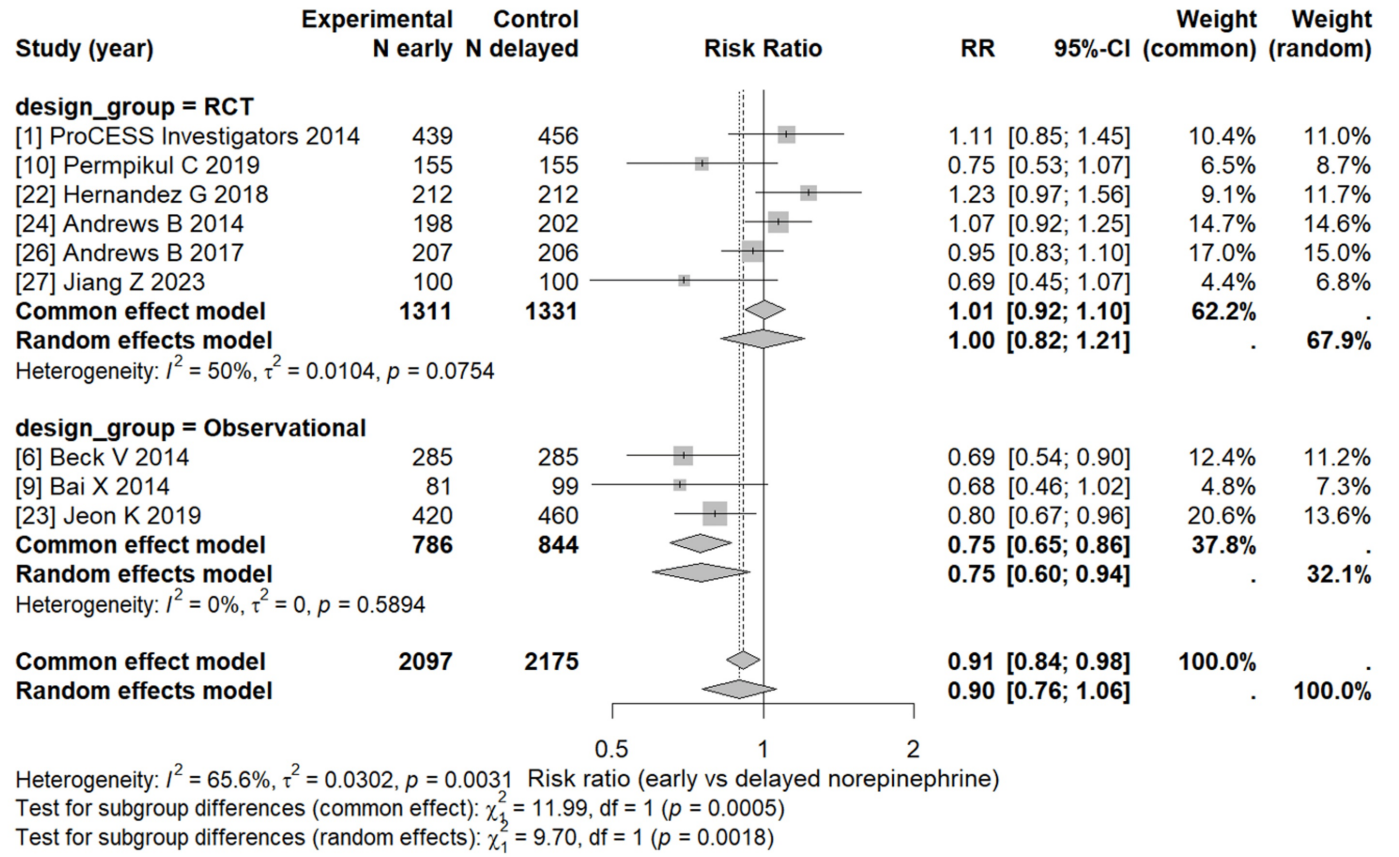
Subgroup meta-analysis by study design A forest plot comparing mortality outcomes between early and delayed norepinephrine initiation, stratified by study design (randomized controlled trials vs observational studies). Squares represent study-specific risk ratios, with diamond markers denoting pooled subgroup estimates.

Further, Figure 3 explores the influence of the study design on treatment outcomes. This analysis provides evidence suggesting potential benefits of early norepinephrine administration while highlighting variability across study types and patient populations.

In the subgroup analysis comparing early versus delayed norepinephrine initiation, randomized controlled trials showed no significant difference in mortality between the two groups (pooled RR = 1.00; 95% CI 0.82-1.21; I² = 50%). In contrast, observational studies indicated a significant reduction in mortality with early administration (pooled RR = 0.75; 95% CI 0.60-0.94; I² = 0%). When all studies were combined, the overall pooled estimate suggested a non-significant trend favoring early norepinephrine (RR = 0.90; 95% CI 0.76-1.06; I² = 65.6%). A significant difference was observed between study designs (p = 0.002), suggesting that the type of study may partly explain the observed heterogeneity.

Study findings

This systematic review showed that the early administration of norepinephrine is a key factor that positively influences the treatment of patients with septic shock by reducing mortality, stabilizing hemodynamics, and decreasing organ dysfunction. Randomized and observational studies are consistent in evidence of the benefits of early vasopressor use. The ANDROMEDA-SHOCK trial has shown that goal-oriented resuscitation based on the perfusion parameters resulted in higher survival and shock-related response than the usual care [22]. Likewise, Jeon et al. [23] have found that the time of starting vasopressors was correlated with the improvement of clinical outcomes in Korean ICUs.

The resource-limited studies, such as Andrews et al. in Zambia, stressed that early resuscitation guidelines lowered the mortality rate despite the infrastructural limitations [24,26]. Furthermore, Jiang et al. discovered that the restrictive form of fluid resuscitation coupled with earlier norepinephrine enhanced the functioning of the liver, spleen, and kidney, and survival percentages [27].

Physiological studies point to the significance of fluid and vasopressor therapy balancing. Shi et al. and Xu et al. demonstrated that the early norepinephrine reinstated the perfusion pressure successfully and decreased the excessive administration of fluids [28, 29]. A personalized approach is emerging with supporting evidence, such as Monnet et al., who found that the timing of norepinephrine administration was customized to each patient based on hemodynamic response [30].

Additionally, adjunctive therapies like early vasopressin have the potential to optimize the outcomes when used in conjunction with norepinephrine [31]. Early initiation of vasopressors is now a primary aspect of clinical guidelines of septic shock management [28].

Discussion

This meta-analysis assessed nine studies to determine whether early norepinephrine initiation reduces mortality in septic shock. The random-effects model showed a non-significant 10% relative reduction in mortality with early administration (RR = 0.90; 95% CI: 0.76-1.06; p = 0.18), while the common-effect model indicated a modest but significant benefit (RR = 0.91; 95% CI: 0.84-0.98; p = 0.016). Moderate heterogeneity was observed (I² = 65.6%), reflecting differences in study design and populations. Subgroup analysis revealed no mortality benefit in randomized controlled trials (RR = 1.00; 95% CI: 0.82-1.21) but a significant reduction in observational studies (RR = 0.75; 95% CI: 0.60-0.94; p = 0.002 for subgroup difference). These findings suggest that early norepinephrine use may improve outcomes by stabilizing perfusion earlier, as supported by physiologic rationale [12-14], although consistent survival benefit across trials remains unproven. Further high-quality randomized studies are warranted to confirm its optimal timing in septic shock management.

Septic shock has continued to be the number one cause of mortality in the critical care setup, with its prevalence in both high and low-income settings increasingly becoming a point of concern in the world today [1,2,6]. According to epidemiological data, sepsis is a leading cause of almost 11 million deaths each year, and there is an urgent need to identify and intervene as soon as possible [4].

Nevertheless, the ProCESS trial showed that protocolized early care did not show any better results in terms of mortality reduction as compared to usual care, which implies that general sepsis management has improved and reduced differences [1]. Equally, the CLASSIC study was in favor of a more restrictive approach to fluid administration post the initial resuscitation and showed no adverse effect in relation to liberal strategies [3]. These results highlight the need to pay more attention to the personalized fluid management, without either under-resuscitation or excess of fluid, which deteriorates the results. Also, perfusion-guided resuscitation had better survival and less dysfunction of the organs, which further enhanced precision-based early management [21].

According to the CENSER trial, the use of norepinephrine in the initial hour (less than 1 hour) resulted in a faster septic shock arrest and reduced short-term death rates [10]. On the same note, early treatment within the first two hours of diagnosis was linked to much reduced mortality compared to delayed administration [9]. These results are further substantiated by observational studies that found out that an extremely early initiation of norepinephrine resulted in better survival and decreased the number of organ failures [11]. On the other hand, it was also shown that later use of vasopressors was also associated with increased 30-day mortality and especially when hypotension was unresolved prior to the use of vasopressors [6].

In addition to norepinephrine, eventual adjunctive vasopressors and customized measures have garnered interest in striving to maximize the results in sepsis shock. Time and choice of vasopressors used were also studied, with norepinephrine being the preferred agent since its safety and efficacy profile is better [28]. Recent data confirms an individualized strategy, which contends that the timing of norepinephrine relies upon real-time hemodynamic variables and not protocols [30].

Furthermore, researchers discovered that concomitant use of adjunctive vasopressin can be used at the beginning of the treatment to sustain perfusion pressure and to reduce the use of high doses of catecholamines [31]. Lastly, methodological rigor in such studies is still essential, and standardized quality assessment instruments, including RoB 2 and Newcastle-Ottawa Scal,e have commonly been utilized to assess the validity of the trial and reduce bias [20, 21].

Limitations and recommendations

There are a number of limitations that should be acknowledged.First, this review included only English-language studies published between January 2010 and May 2025, which may have resulted in the exclusion of relevant studies published earlier or in other languages.** **Second, the grey literature was not covered, which included conference proceedings, theses, and unpublished data, which might have excluded new evidence in an ongoing trial. Third, differences in study design, the definition of early norepinephrine initiation, and differences in clinical settings led to heterogeneity, which restricts direct comparisons among studies.

Based on this study's findings, which show a promising mortality benefit for early norepinephrine use, we strongly recommend that societies such as the Society of Critical Care Medicine,** **the Infectious Diseases Society of America,** ** and the American College of Emergency Physicians, along with major academic health systems, take a forefront role in transforming this evidence into actionable clinical practice. There is a need to develop a unified clinical pathway that advocates for a standardized timing (commence within one hour) benchmark for vasopressor initiation, where feasible, to cut through the current ambiguity. In addition, we must champion and fund large multinuclear RCTs to resolve the mortality signal discrepancy seen between observational studies and RCTs. Finally, integrating exclusive training modules on the rationale for early hemodynamic stabilization into Continuing Medical Education (CME) and critical care fellowship curricula is an important step to ensure this potential best practice is understood and implemented effectively at the bedside by the next generation of intensivists.

## Conclusions

In conclusion, this review indicates that early norepinephrine administration in septic shock is associated with reduced mortality, faster shock resolution, and improved organ function compared to delayed use. Combining early vasopressor therapy with careful fluid resuscitation optimizes hemodynamic stability and minimizes complications such as fluid overload. While discrepancies exist across studies, these findings support timely, evidence-based intervention and highlight the need for large multicenter trials to establish standardized guidelines for vasopressor timing.

Further research is needed to clarify optimal dosing and timing protocols across diverse patient populations. As clinical evidence evolves, more precise guidelines may improve clinician confidence and patient outcomes in septic shock management.
